# Supplementary material for: DNA targeting by compact Cas9d and its resurrected ancestor
Source: Nat Commun. 2025 Jan 7;16:457. doi: 10.1038/s41467-024-55573-4 (PMC11706934; doi:10.1038/s41467-024-55573-4)

Supplementary Information

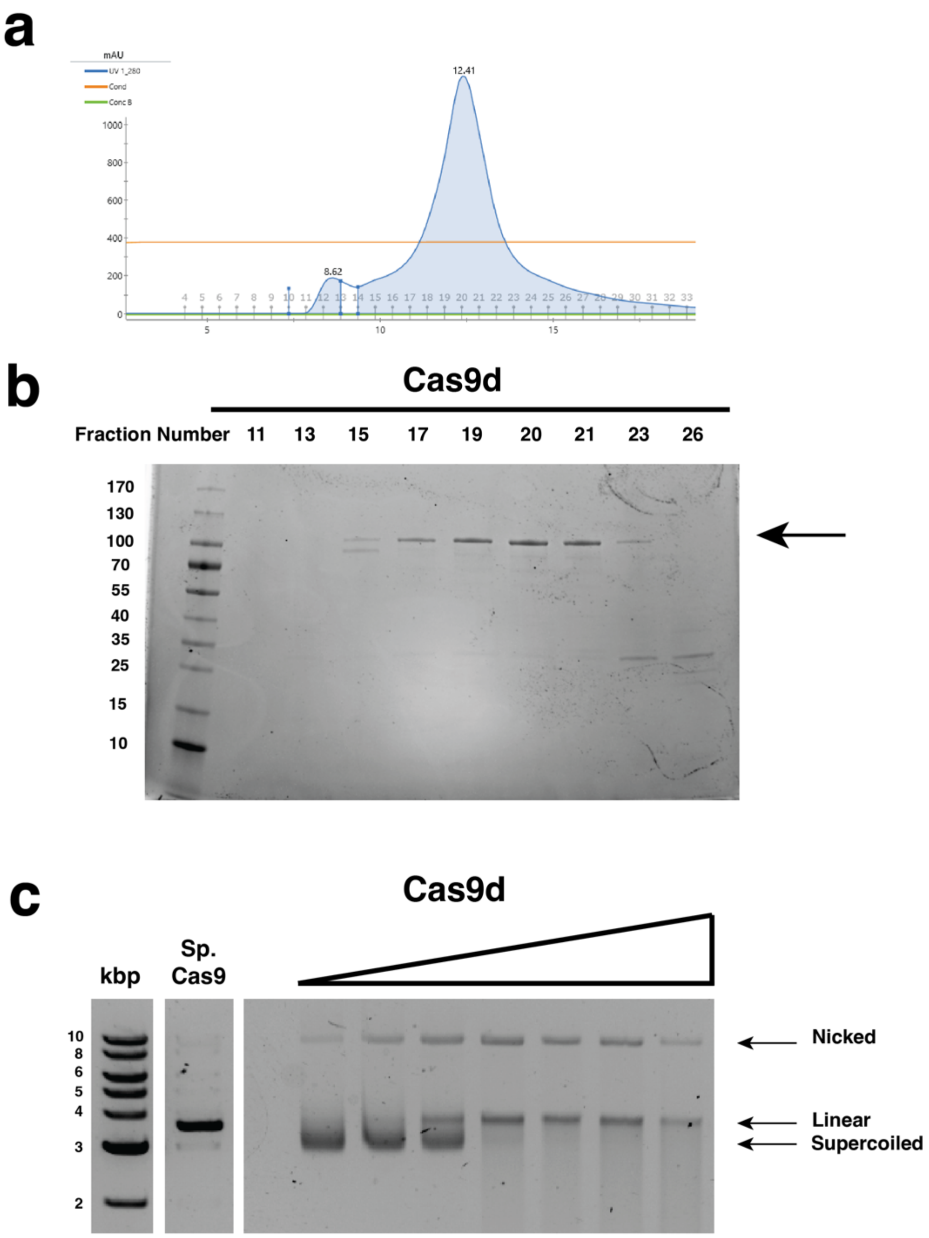

Supplementary Fig. 1 Purification and in vitro cleavage for Cas9d. a, Cas9d Size Elution Chromatography (SEC) chromatogram. **b,** Cas9d PAGE gel. **c,** Cas9d Plasmid in vitro cleavage assay with purified RNP and no sgRNA supplementation.

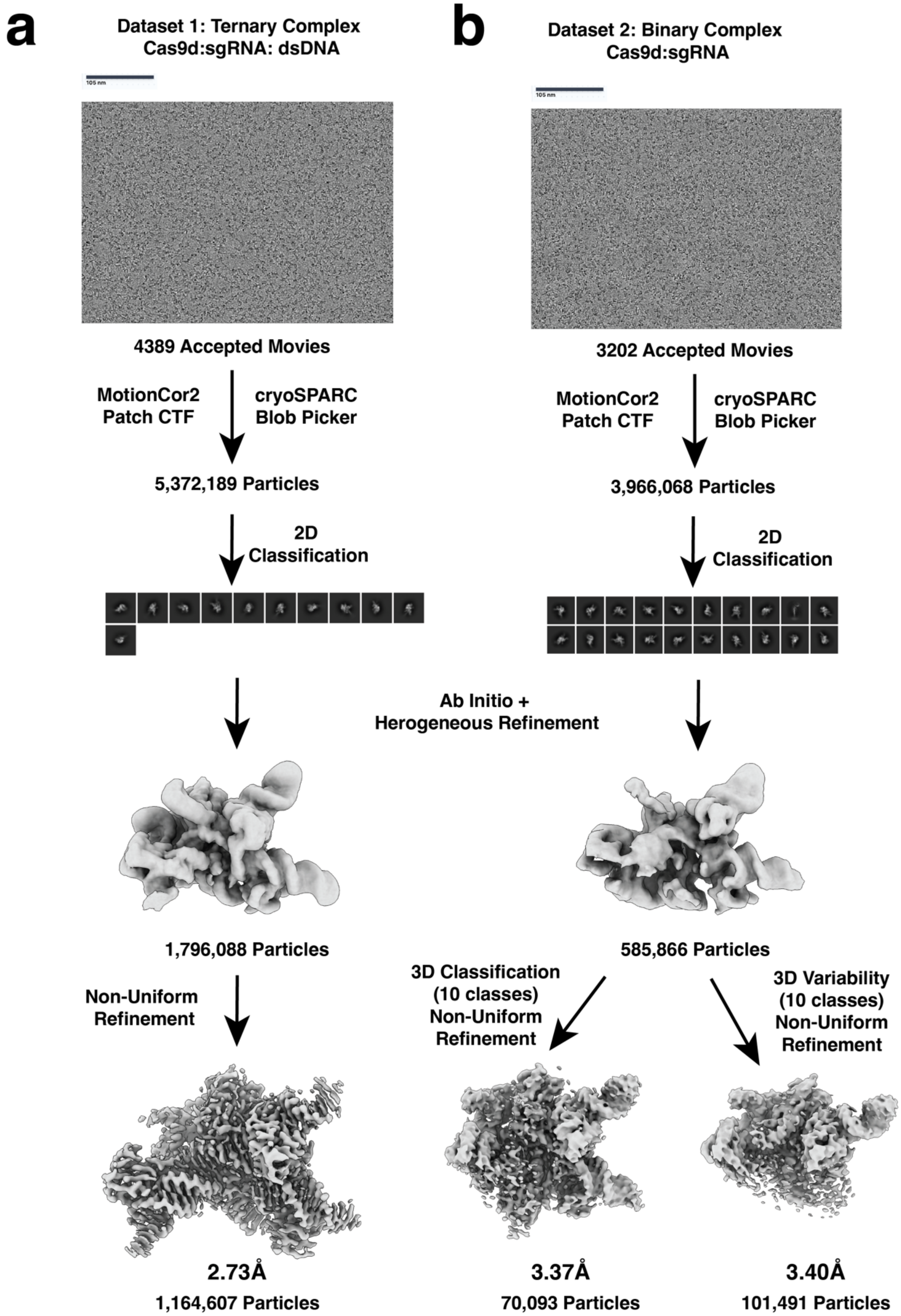

Supplementary Fig. 2. Cryo-EM dataset processing pipeline. a, Processing pipeline for 20bp R-loop complex. **b,** Processing pipeline for 6bp seed and binary complexes.

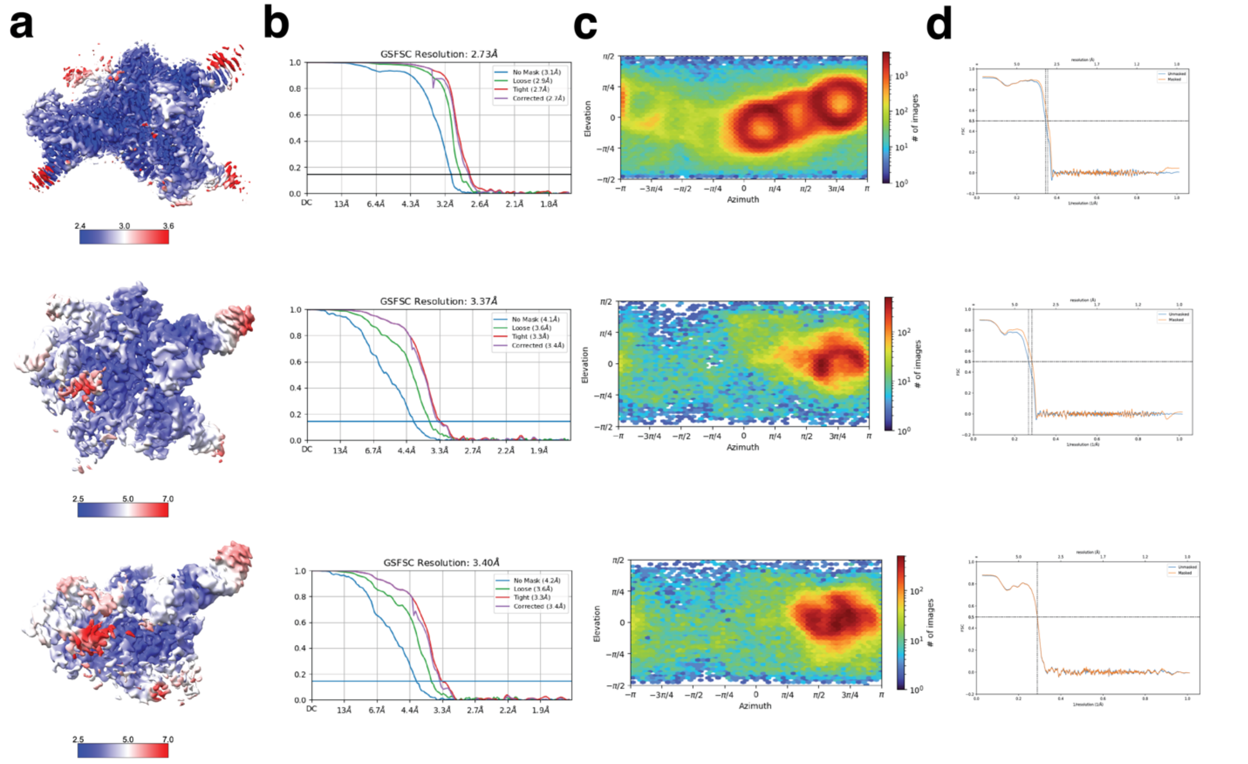

Supplementary Fig. 3. Cas9d cryo-EM data analysis and quality control. a, Sharpened maps colored to local resolution with gold-standard FSC curves for all three Cryo-EM reconstructions. From top to bottom: 20bp R-loop complex, 6-bp seed conformation and binary complex. **b,** All resolutions reported are for sharpened maps at an FSC threshold of 0.143. **c,** Euler diagrams show orientation distribution of Cryo-EM reconstructions. **d,** Map to model FSCs are shown for all three Cryo-EM reconstructions

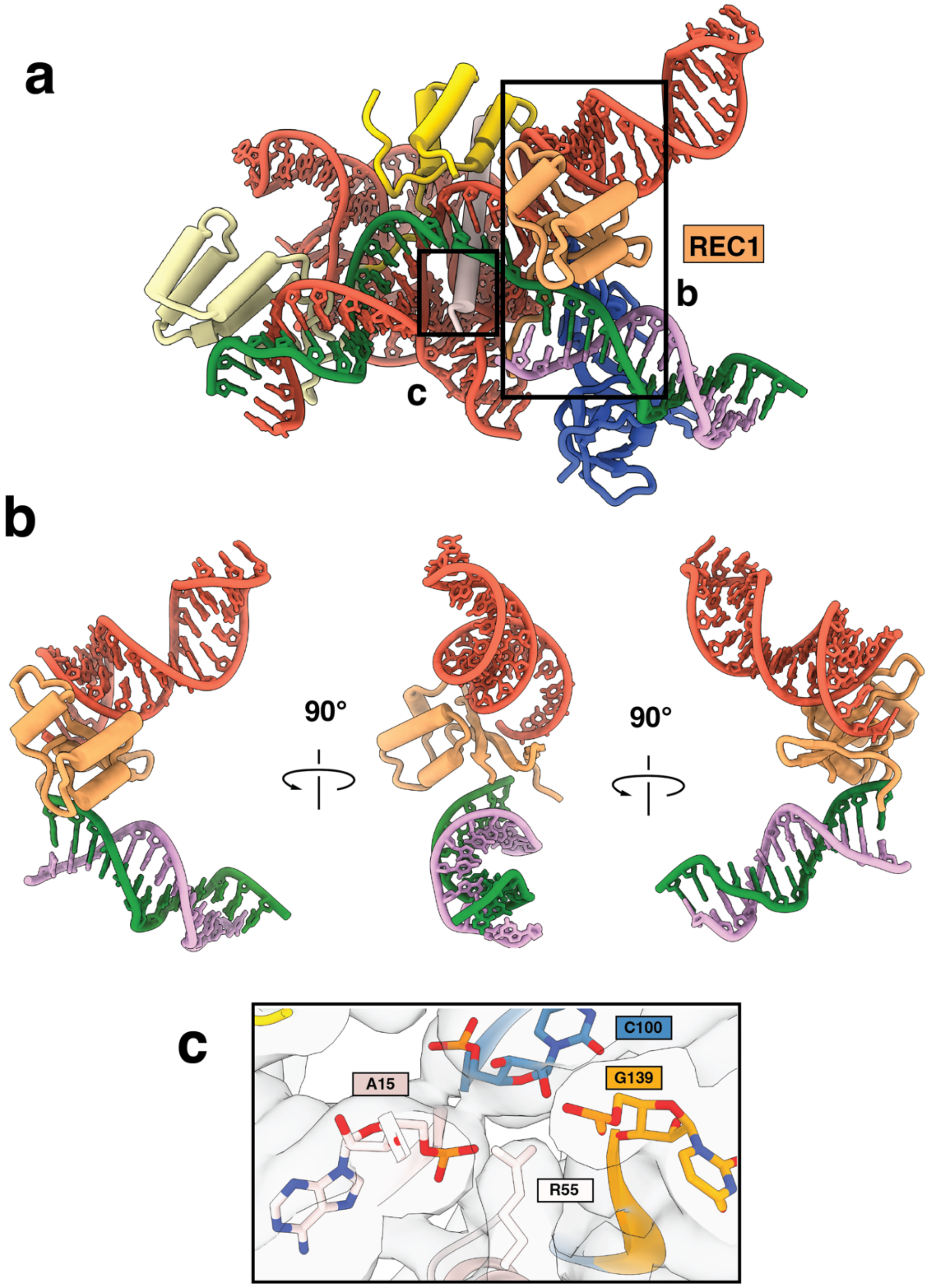

Supplementary Fig. 4. Overview of Cas9d REC1 domain. a, Overview of REC1 domain wedged between the sgRNA and the target DNA **b,** Front, middle and back views of the REC1 domain show that a REC1 loop coordinates both the sgRNA and the DNA target and non-target strands. **c,** R55 stabilized the negative charge of the phosphate backbones of the sgRNA.

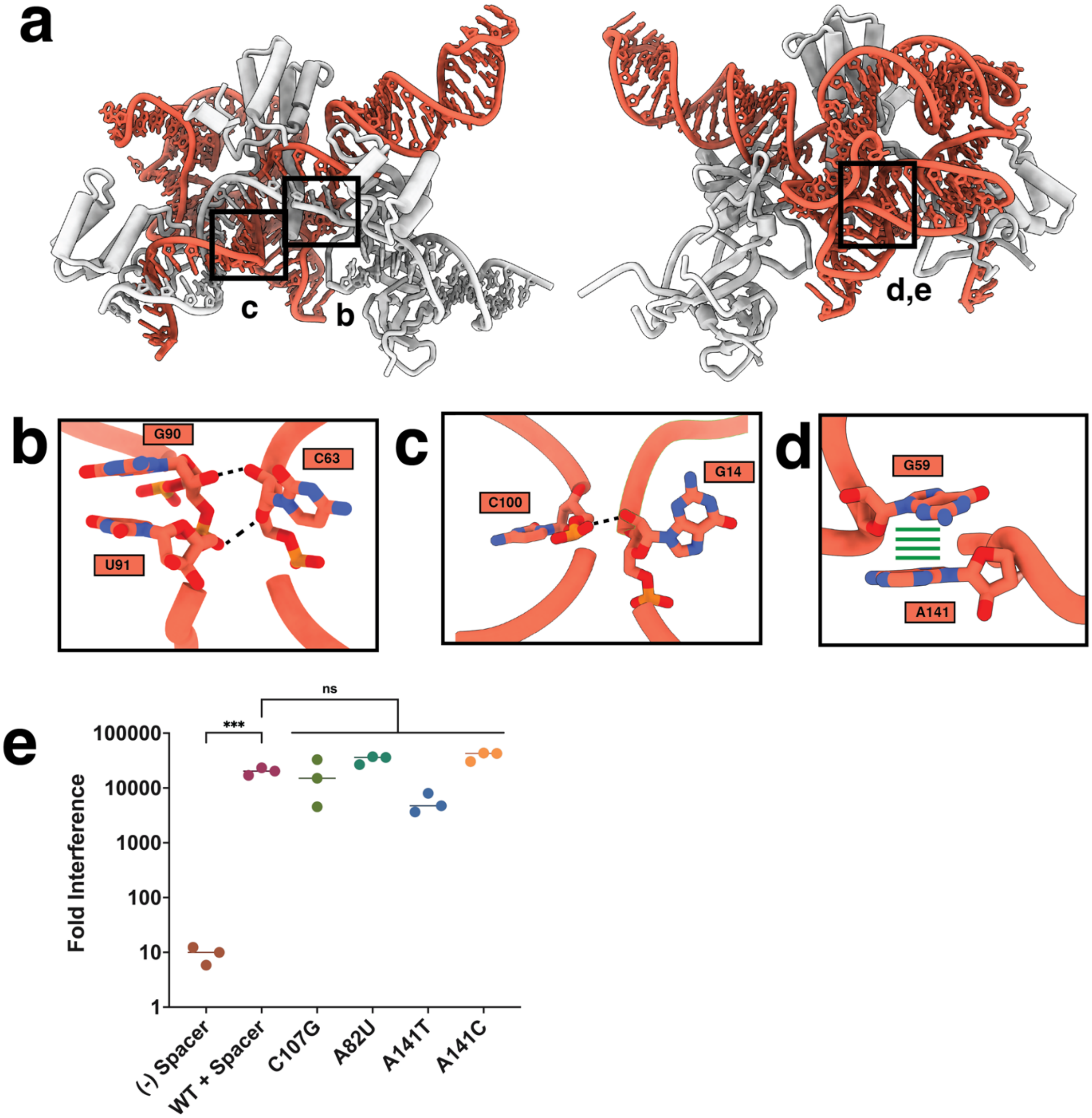

Supplementary Fig. 5. Overview of sgRNA stabilization interactions. a, Overall structure (front and back) of the sgRNA (red) and the effector (grey). **b,** Long range stabilization interaction between the phosphate backbones of C93, U91, and G90. **c,** Long range stabilization interaction between the phosphate backbones between C100 and G14, showing spacer stabilization by the sgRNA. **d,** Base stacking between G59 and A141 stabilize the P1 and P3 region folding. **e,** Cas9d nucleic acid targeting efficiency after purine to pyrimidine mutations in the RNA triplex and the P3.1 region. All experiments were done in biological triplicates. Statistical significance is evaluated via two-sided unpaired t-tests; ***: $p < 0.001$.

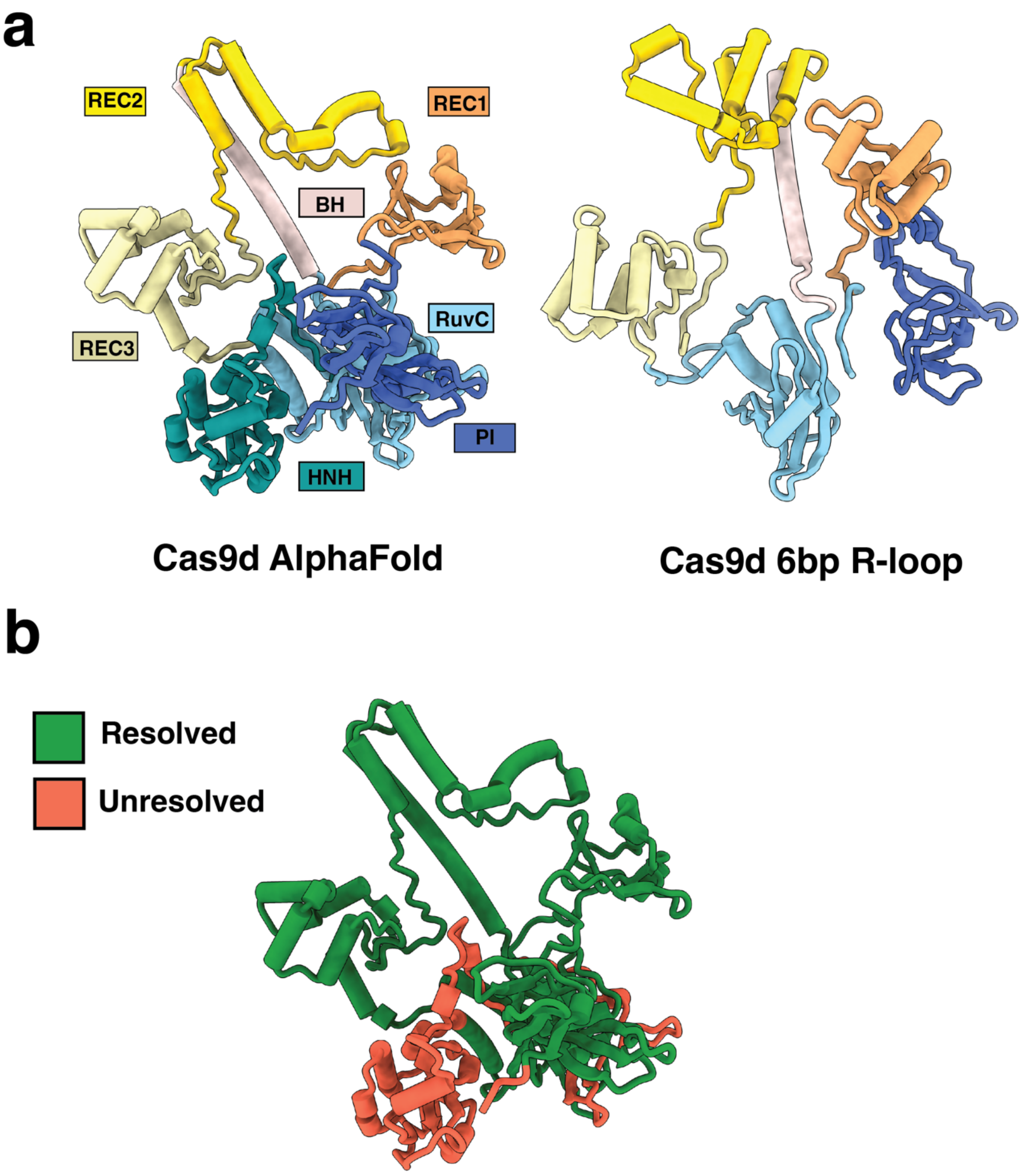

Supplementary Fig. 6: Comparison between Cas9d cryo-EM structure and AlphaFold. a, Cas9d predicted structure using Alphafold compared to the Cas9d seed 6-bp R-loop model (no nucleic acids shown). Clear differences in REC1 and REC2 positioning are observed. **b,** Cas9d map colored by resolved vs unresolved regions. Coloring clearly shows that HNH is not resolved in the 6-bp R-loop structure.

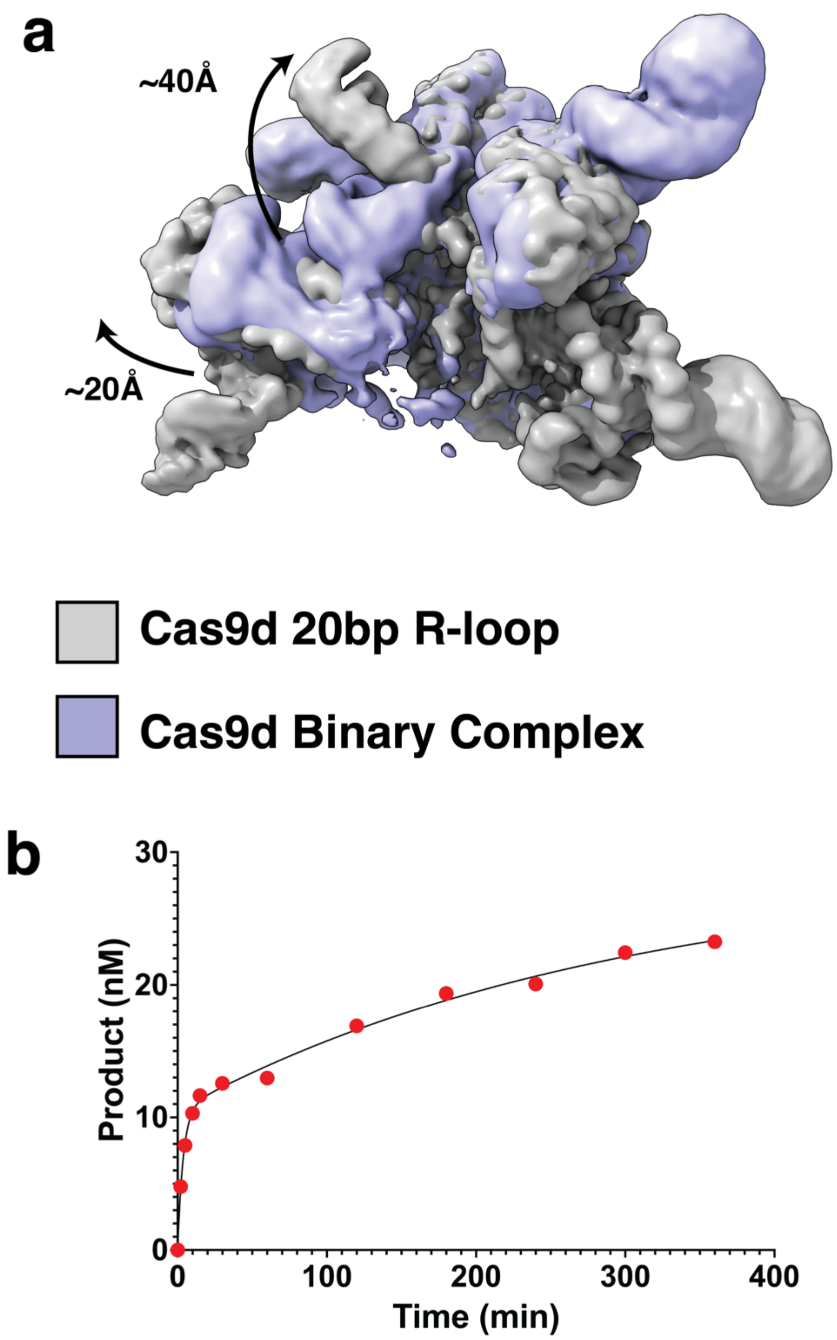

Supplementary Fig 7. Overview of Cas9d REC domain rearrangements. Low-pass filtered unsharpened maps for the 20bp R-loop complex (grey) and binary complex (magenta) show clear REC2 and REC3 domain rearrangements. **b,** Cas9d turnover assay show slow rate of Cas9d turnover *in vitro* with a rate of $3.4 \times 10^{-3} min^{-1}$. The concentration of Cas9d is constant at 10 nM.

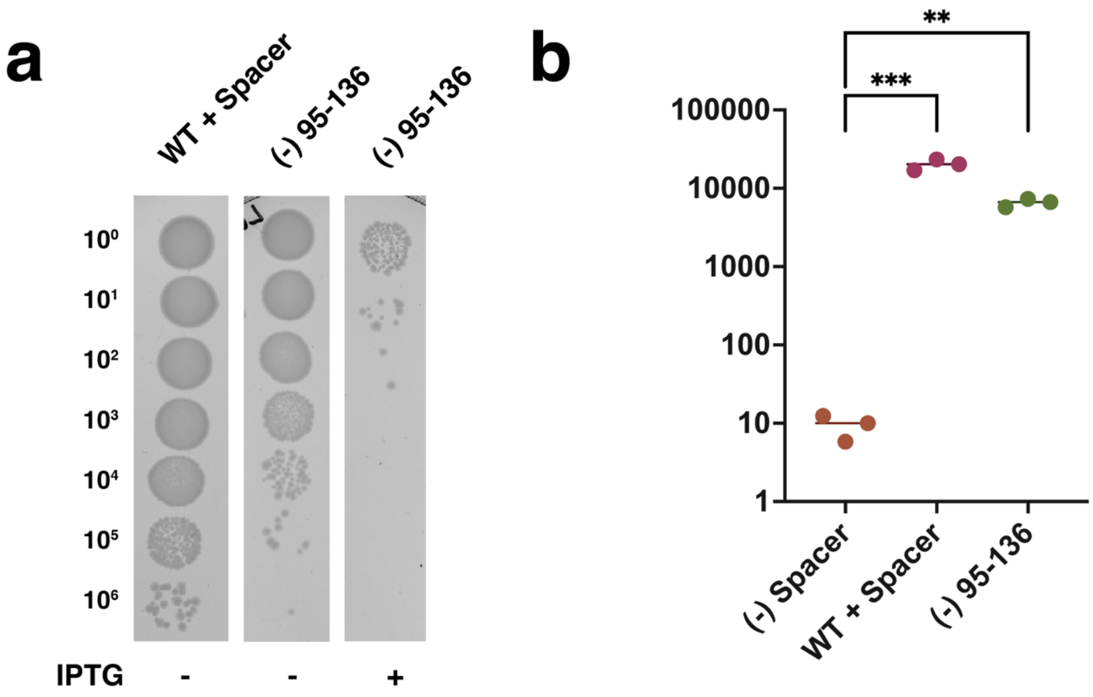

Supplementary Fig. 8. Cas9d activity with (-) 95-136 deletion. a, Representative image of DNA targeting assays performed using Cas9d (-) 95-136 REC2 deletion compared to WT Cas9d. **b,** Quantification of DNA targeting activity by Cas9d (-) 95-136 REC2 deletion shows that Cas9d retains activity levels similar to WT Cas9d. All experiments were done in biological triplicates. Statistical significance is evaluated via two-sided unpaired t-tests; ** : $p < 0.01$ ***: $p < 0.001$.

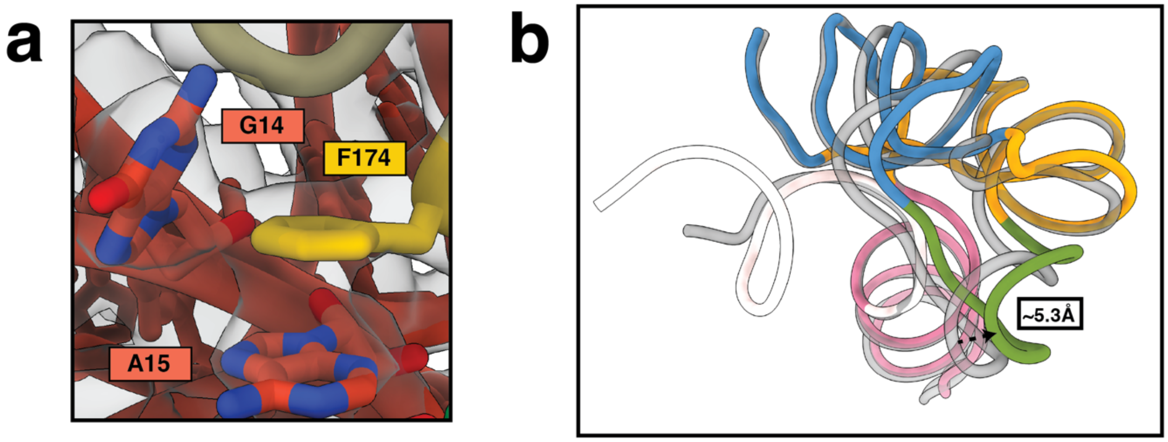

Supplementary Fig. 9. Guide rearrangements between structures. a, sgRNA rearrangement between the seed complex (grey) and 20bp complex (in color) shows a 5.3Å rearrangement of the P4 hairpin. **b,** F174 base stacks between A15 and G14 in the seed complex, stabilizing this conformation.

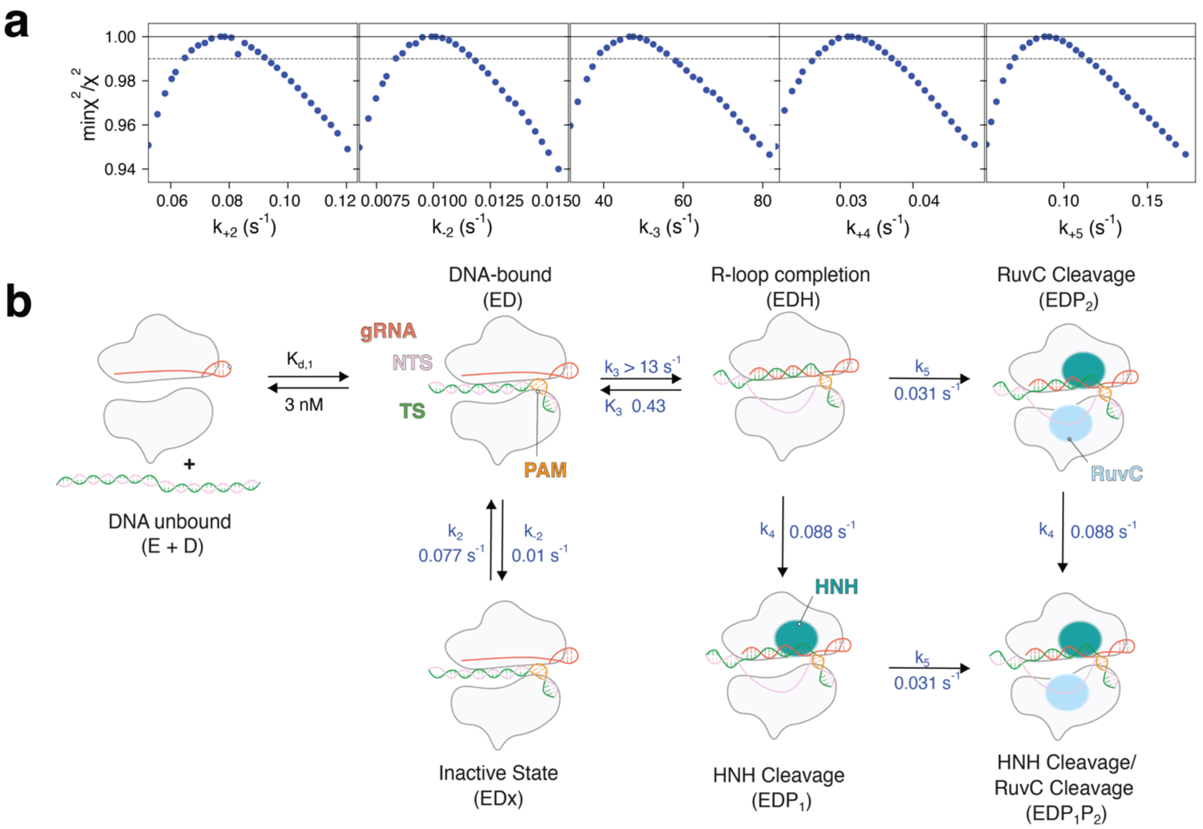

Supplementary Fig. 10. Confidence contour analysis and kinetic model for Cas9d cleavage. a, Confidence contour plots for fits to the model in (**b**). Contours show that with R loop formation locked at its lower limit, all parameters are well constrained by the data. The 95% confidence interval is given by the dashed line, calculated at a 0.99 min χ^2/χ^2 threshold based on the number of data points and parameters in the fitting. **b,** Kinetic model for target acquisition and cleavage by Cas9d. Rate constants derived from the fitting of kinetic data in Fig. 6a-e are shown in blue. The equilibrium constant for DNA binding (K_{d1}) was locked in the fitting at a reasonable value as it was not defined by the data and is shown in black.

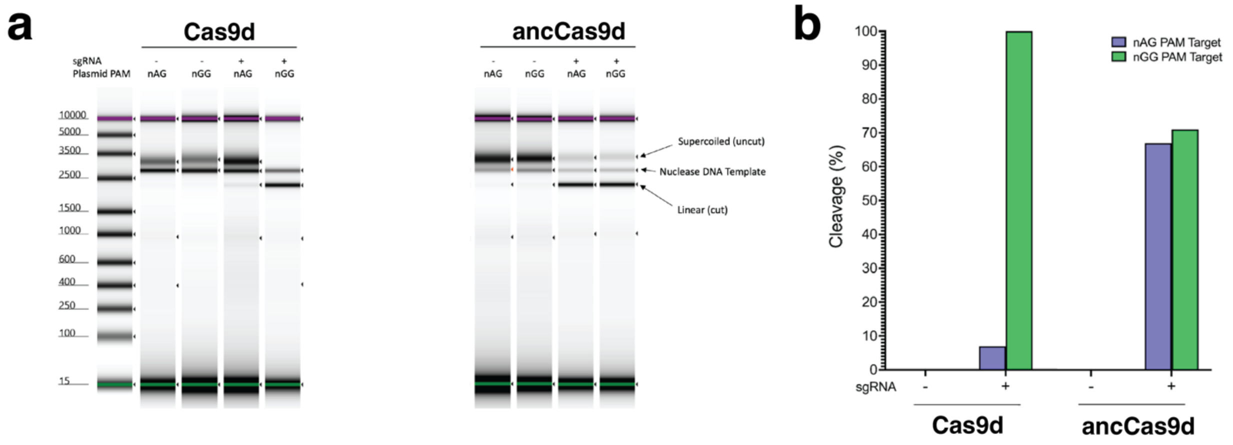

Supplementary Fig. 11. *In vitro* Cas9d PAM targeting efficiency by AncCas9d. a, In vitro plasmid cleavage comparison between Cas9d and ancCas9d (34-29). **b,** Quantification of in vitro cleavage data shows improved NAG PAM targeting for ancCas9d. Representative of three independent experiments.

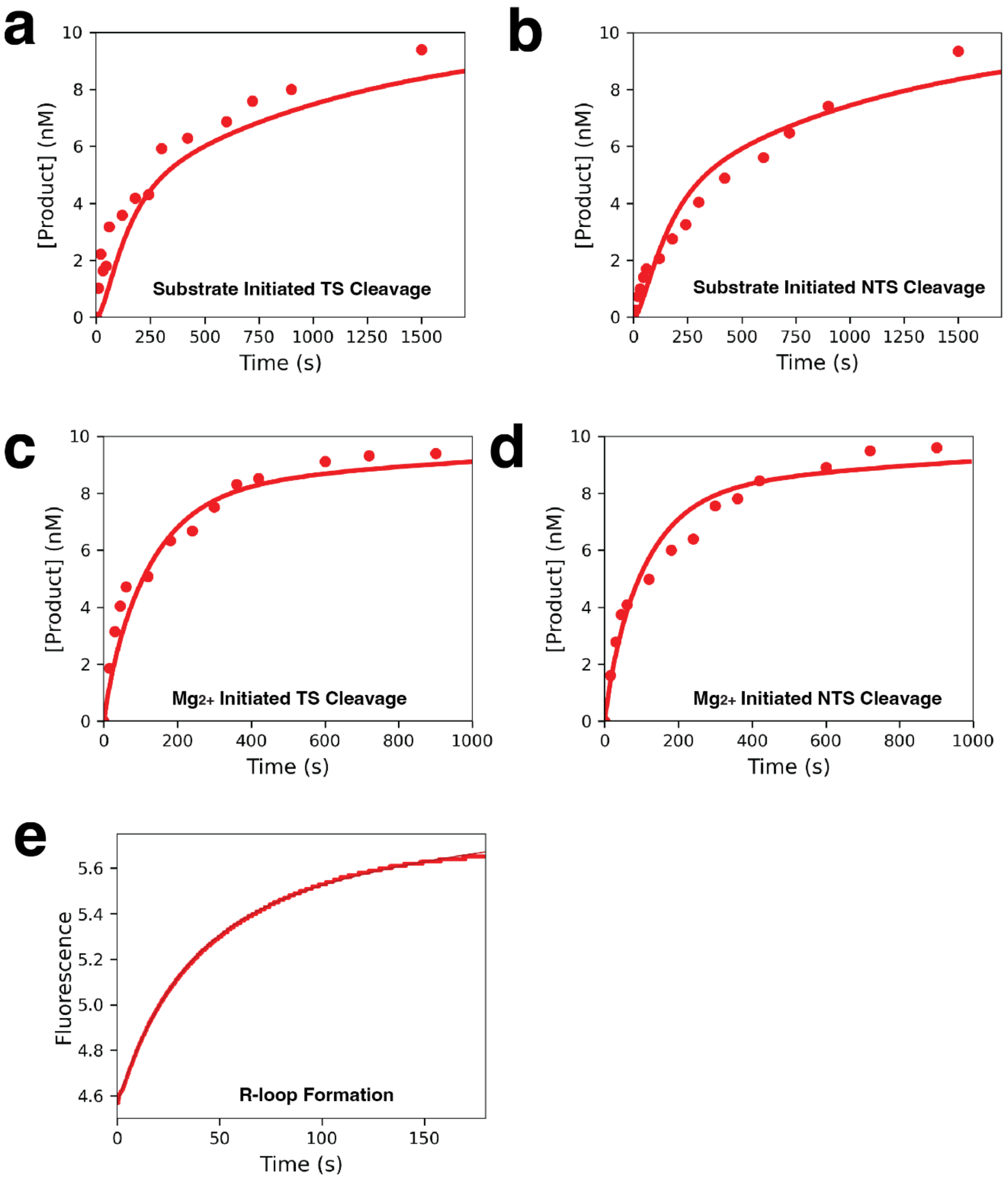

Supplementary Fig. 12 Kinetic characterization of ancCas9d reveals ancCas9d preferentially partitions into the full R-loop Complex. a, Substrate initiated target strand cleavage. **b,** Substrate initiated non-target strand cleavage. **c,** Mg^{2+} initiated target strand cleavage. **d,** Mg^{2+} initiated non-target strand cleavage. **e,** R-loop formation assays for Cas9d using a tC° Substrate.

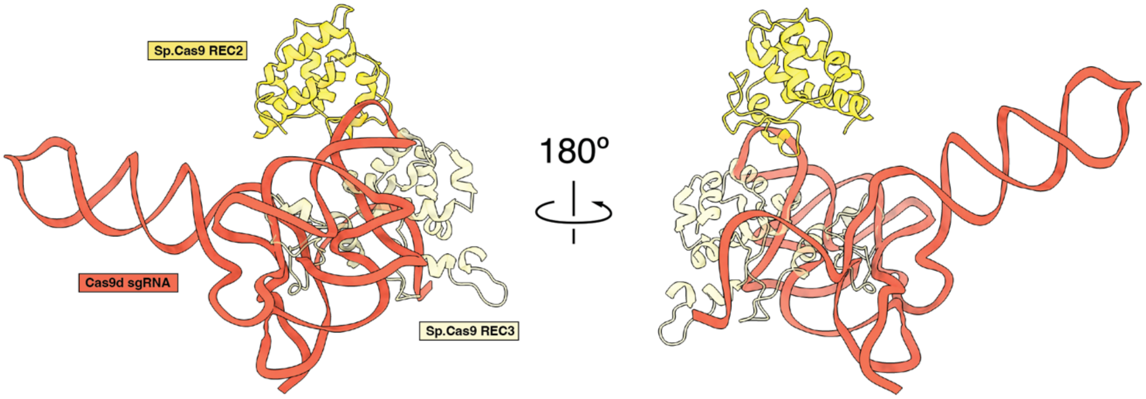

Supplementary Fig. 13. Cas9d sgRNA occupies the same positions as Sp.Cas9 REC2 and REC3 domains. Structures were aligned using the R-loop as reference. The P4 sgRNA region occupies the same position as REC2. P2 and P4 occupy similar positions to REC3.

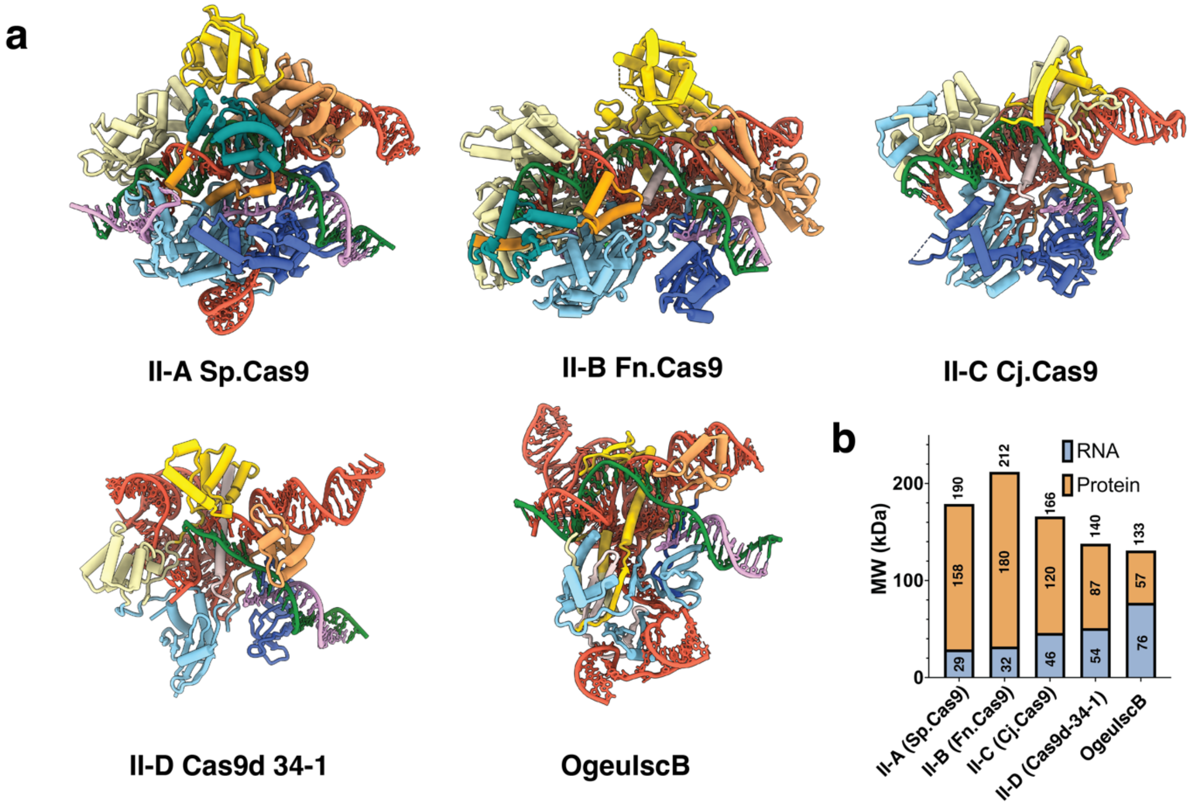

Supplementary Fig. 14. Comparison between Cas9d and other homologs. a, Comparison between Cas9d, IscB, and other type II structures previously solved. In order, Sp.Cas9, Fn.Cas9, Cj.Cas9, Cas9d , OgeuIscB. Target Strand (TS) forest green; Non-Target Strand (NTS) plum; RuvC sky blue; Bridge Helix misty rose; REC2 sandy brown; REC3 gold; HNH dark cyan; REC1 sandy brown; PI royal blue. Colors are maintained throughout except for IscB where its PLMP domain is colored in gold instead of REC3. **b,** Molecular Weight comparison of the sgRNA vs the effector for OgeuIscB, Cas9d, Sp.Cas9, Fn.Cas9, and Cj.Cas9.

Supplementary Table 1. Cryo-EM data collection, refinement and validation statistics.

	Cas9d 20bp R-loop Complex (EMDB-xxxx) (PDB xxxx)	Cas9d Seed Complex (EMDB-xxxx) (PDB xxxx)	Cas9d Binary Complex (EMDB-xxxx) (PDB xxxx)
**Data collection and processing**			
Magnification	150kx	150kx	150kx
Voltage (kV)	300	300	300
Electron exposure (e–/Å^2)	80	80	80
Defocus range (μm)	1.5-2.5	1.5-2.5	1.5-2.5
Pixel size (Å)	0.8332	0.8332	0.8332
Symmetry imposed	C1	C1	C1
Initial particle images (no.)	1,796,088	585866	585866
Final particle images (no.)	1,194,704	70043	101411
Map resolution (Å)	2.71	3.37	3.40
FSC threshold. \`.	0.143	0.143	0.143
Map resolution range (Å)	2.4-4.0	2.5-7.0	2.5-7.0
**Refinement**			
Initial model used (PDB code)	NA	NA	NA
Model resolution (Å)	NA	NA	NA
FSC threshold			
Model resolution range (Å)	NA	NA	NA
Map sharpening *B* factor (Å^2)	144	147.7	156.5
Model composition			
Non-hydrogen atoms	7256	7886	5706
Protein residues	389	525	339
Nucleotide residues	192	170	137
Ligands	NA	NA	NA
B factors (Å^2)			
Protein	43.22	112.94	106.55
Nucleotide	45.47	113.51	113.52
Ligand	NA	NA	NA
R.m.s. deviations			
Bond lengths (Å)	0	0	0
Bond angles (°)	9	9	9
Validation			
MolProbity score	1.23	1.56	1.62
Clashscore	4.61	6.08	6.53
Poor rotamers (%)	0.57	0.86	0.95
Ramachandran plot			
Favored (%)	98.95	95.71	95.12
Allowed (%)	1.05	4.29	4.88
Disallowed (%)	0	0	0

Supplementary Figure Source Data

Supplementary Fig. 1. Source Data.

Uncropped gel image for panel (b).

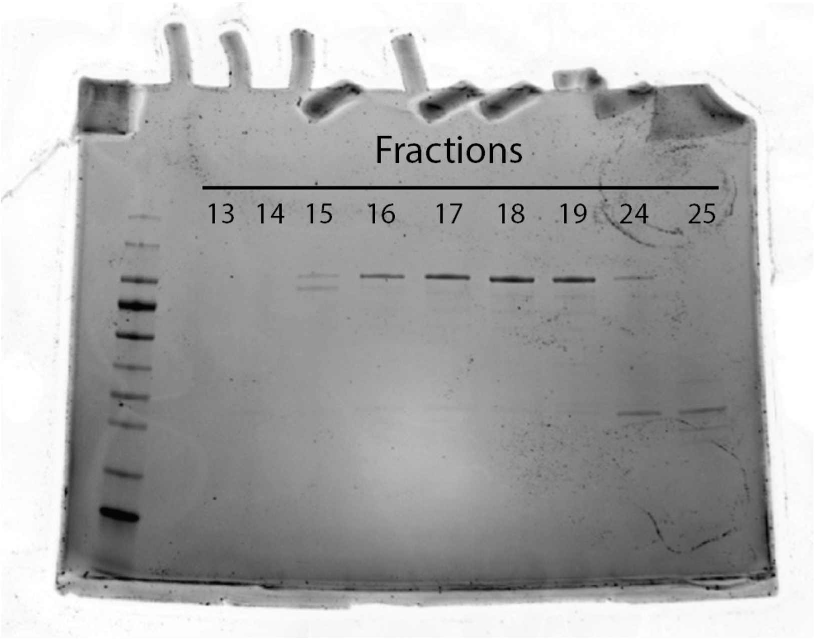

Uncropped gel image for panel (c).

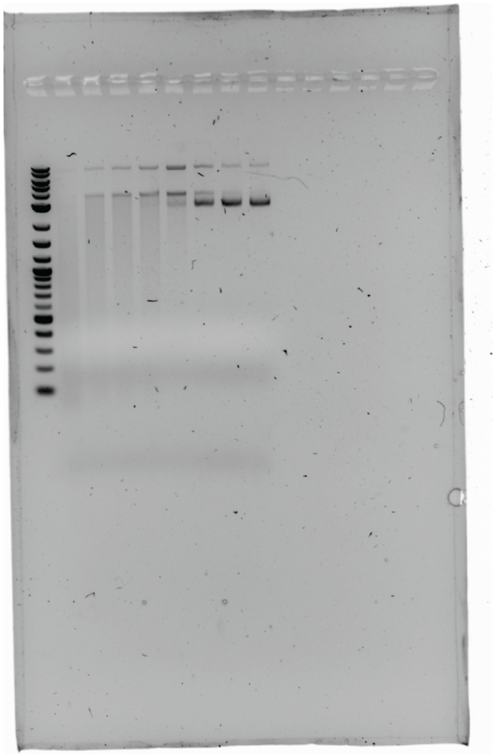

Supplementary Fig. 9 Source Data.

Uncropped plate images for panel (a).

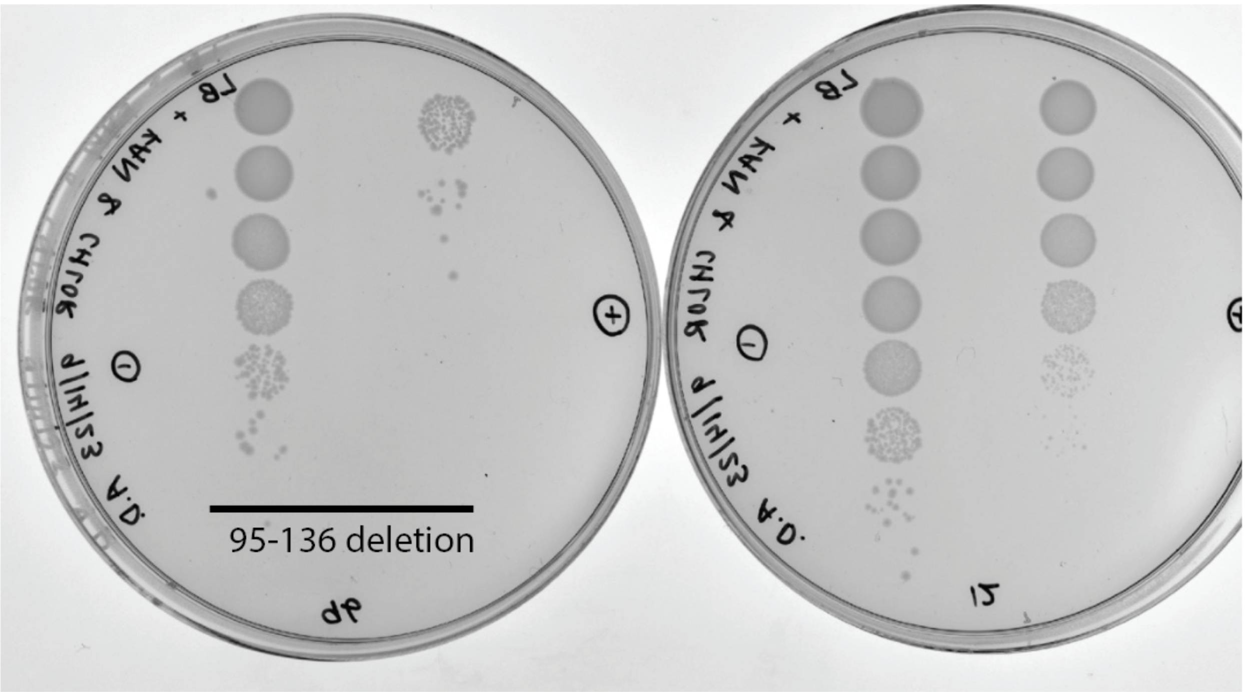

Supplement: Supplementary file 1 — Supplementary Information [file 41467_2024_55573_MOESM1_ESM.pdf]
