# Supplementary material for: DNA targeting by compact Cas9d and its resurrected ancestor
Source: Nat Commun. 2025 Jan 7;16:457. doi: 10.1038/s41467-024-55573-4 (PMC11706934; doi:10.1038/s41467-024-55573-4)
Supplement: Supplementary file 2 — Reporting Summary [file 41467_2024_55573_MOESM2_ESM.pdf]

nature portfolio

Corresponding author(s): David W. Taylor

Last updated by author(s): Nov 18, 2024

Reporting Summary

Nature Portfolio wishes to improve the reproducibility of the work that we publish. This form provides structure for consistency and transparency in reporting. For further information on Nature Portfolio policies, see our Editorial Policies and the Editorial Policy Checklist.

Statistics

For all statistical analyses, confirm that the following items are present in the figure legend, table legend, main text, or Methods section.

n/a	Confirmed	
☐	☒	The exact sample size (*n*) for each experimental group/condition, given as a discrete number and unit of measurement
☐	☒	A statement on whether measurements were taken from distinct samples or whether the same sample was measured repeatedly
☐	☒	The statistical test(s) used AND whether they are one- or two-sided<br>*Only common tests should be described solely by name; describe more complex techniques in the Methods section.*
☒	☐	A description of all covariates tested
☒	☐	A description of any assumptions or corrections, such as tests of normality and adjustment for multiple comparisons
☐	☒	A full description of the statistical parameters including central tendency (e.g. means) or other basic estimates (e.g. regression coefficient) AND variation (e.g. standard deviation) or associated estimates of uncertainty (e.g. confidence intervals)
☐	☒	For null hypothesis testing, the test statistic (e.g. *F*, *t*, *r*) with confidence intervals, effect sizes, degrees of freedom and *P* value noted<br>*Give P values as exact values whenever suitable.*
☒	☐	For Bayesian analysis, information on the choice of priors and Markov chain Monte Carlo settings
☒	☐	For hierarchical and complex designs, identification of the appropriate level for tests and full reporting of outcomes
☒	☐	Estimates of effect sizes (e.g. Cohen's *d*, Pearson's *r*), indicating how they were calculated

Our web collection on statistics for biologists contains articles on many of the points above.

Software and code

Policy information about availability of computer code

Data collection	Data were collected on an FEI Titan Krios 300V Transmission Electron Microscope (Thermo Scientific) with a Gatan BioContinuum Imaging Filter and a K3 direct electron detector. Data were collected on Serial EM v3.9 with a 0.832Å pixel size and a defocus range of -1.5 to -2.5um with a total exposure time of 10s in a total accumulated dose of 40e/A2. Motion correction, contrast transfer function (CTF) estimation and particle picking was carried out in croySPARC live v4.0. A total of 4389 movies were accepted at -30° tilt for the 20-bp R-loop complex. 3202 movies were collected for the binary and 6-bp R-loop structures.
Data analysis	Subsequent cryo-EM data was processed using cryoSPARC v3.2.The initial backbone for the Cas9d 20-bp R-loop map was built using an automatic atomic model builder program, ModelAngelo (Cosmic2). The output from this software showed a relatively well-aligned RNA backbone as well as the alpha helices from the protein. The output was used as a fiducial for de-novo model building using a combination of coot v1.0 and ChimeraX v1.720. Once fully modeled, Isolde v1.4 was used to improve the model to map fit and for rotamer optimization. Multiple iterations of real space refinement implemented within Phenix v1.19 were carried out to improve the model geometry and map fit.

For manuscripts utilizing custom algorithms or software that are central to the research but not yet described in published literature, software must be made available to editors and reviewers. We strongly encourage code deposition in a community repository (e.g. GitHub). See the Nature Portfolio guidelines for submitting code & software for further information.

Data

Policy information about availability of data

All manuscripts must include a data availability statement. This statement should provide the following information, where applicable:

- Accession codes, unique identifiers, or web links for publicly available datasets
- A description of any restrictions on data availability
- For clinical datasets or third party data, please ensure that the statement adheres to our policy

The cryo-EM structures of Cas9d with a 20-bp R-loop, Cas9d with a 6-bp R-loop, and Cas9d:sgRNA binary complex have been deposited into the Electron Microscopy Data Bank (EMDB) with accession codes EMD-43878[https://www.ebi.ac.uk/emdb/EMD-43878], EMD-43760[https://www.ebi.ac.uk/emdb/EMD-43760], EMD-43757[https://www.ebi.ac.uk/emdb/EMD-43757], respectively. The corresponding models have been deposited into the Protein Data Bank (PDB) with accession codes 9AUF[https://www.rcsb.org/structure/9AUF], 8W2Z[https://www.rcsb.org/structure/8W2Z], 8W2S[https://www.rcsb.org/structure/8W2S], respectively. Source data are provided with this paper.

Research involving human participants, their data, or biological material

Policy information about studies with human participants or human data. See also policy information about sex, gender (identity/presentation), and sexual orientation and race, ethnicity and racism.

Reporting on sex and gender	Not applicable
Reporting on race, ethnicity, or other socially relevant groupings	Not applicable
Population characteristics	Not applicable
Recruitment	Not applicable
Ethics oversight	Not applicable

Note that full information on the approval of the study protocol must also be provided in the manuscript.

Field-specific reporting

Please select the one below that is the best fit for your research. If you are not sure, read the appropriate sections before making your selection.

[x] Life sciences [ ] Behavioural & social sciences [ ] Ecological, evolutionary & environmental sciences

For a reference copy of the document with all sections, see nature.com/documents/nr-reporting-summary-flat.pdf

Life sciences study design

All studies must disclose on these points even when the disclosure is negative.

Sample size	The number of particles used were for structural studies was limited by the amount of time that data could be collected. For biochemical experiments, the number of samples analyzed were chosen to show successful replication and to perform statistical significance.
Data exclusions	No data was excluded from this study.
Replication	Experiments that used null-hypothesis testing were the result of three independent biological replicates. Gels and observational experiments are representative of at least two independent experiments. All attempts at replication were successful.
Randomization	No randomization was performed. Randomization was not used in this study because results were observational and did not involve experiments where bias was a concern. Multiple individuals performed each experiment independently.
Blinding	No blinding was performed. Blinding was not used in this study because results were observational and did not involve experiments where bias was a concern. Multiple individuals performed each experiment independently.

Reporting for specific materials, systems and methods

We require information from authors about some types of materials, experimental systems and methods used in many studies. Here, indicate whether each material, system or method listed is relevant to your study. If you are not sure if a list item applies to your research, read the appropriate section before selecting a response.

Materials & experimental systems

n/a	Involved in the study
☒	☐ Antibodies
☐	☒ Eukaryotic cell lines
☒	☐ Palaeontology and archaeology
☒	☐ Animals and other organisms
☒	☐ Clinical data
☒	☐ Dual use research of concern
☒	☐ Plants

Methods

n/a	Involved in the study
☒	☐ ChIP-seq
☒	☐ Flow cytometry
☒	☐ MRI-based neuroimaging

Eukaryotic cell lines

Policy information about cell lines and Sex and Gender in Research

Cell line source(s)	K562 cells were used in cell editing experiments.
Authentication	Cells were purchased from ATCC and cultured according to manufacturer procedures .
Mycoplasma contamination	Cell lines were not tested for Mycoplasma contamination.
Commonly misidentified lines (See ICLAC register)	None used.

Plants

Seed stocks	Not applicable
Novel plant genotypes	Not applicable
Authentication	Not applicable
