# Supplementary material for: DNA targeting by compact Cas9d and its resurrected ancestor
Source: Nat Commun. 2025 Jan 7;16:457. doi: 10.1038/s41467-024-55573-4 (PMC11706934; doi:10.1038/s41467-024-55573-4)
Supplement: Supplementary file 3 — Description of Additional Supplementary Files [file 41467_2024_55573_MOESM3_ESM.pdf]

Supplementary Data File 1. Primers, RNA guides, and DNA substrates.
